# Insight into oral amphiphilic cyclodextrin nanoparticles for colorectal cancer: comprehensive mathematical model of drug release kinetic studies and antitumoral efficacy in 3D spheroid colon tumors

**DOI:** 10.3762/bjoc.19.14

**Published:** 2023-02-13

**Authors:** Sedat Ünal, Gamze Varan, Juan M Benito, Yeşim Aktaş, Erem Bilensoy

**Affiliations:** 1 Department of Pharmaceutical Technology, Faculty of Pharmacy, Erciyes University, 38280, Kayseri, Turkey; 2 Department of Vaccine Technology, Vaccine Institute, Hacettepe University, 06100, Ankara, Turkey; 3 Institute for Chemical Research, CSIC - University of Sevilla, Av. Americo Vespucio 49, 41092, Sevilla, Spain; 4 Department of Pharmaceutical Technology, Faculty of Pharmacy, Hacettepe University, 06100, Ankara, Turkey

**Keywords:** colorectal cancer, camptothecin, 3D spheroid, cyclodextrin, oral nanoparticle, release kinetics

## Abstract

Colorectal cancer (CRC) is the third most diagnosed cancer type globally and ranks second in cancer-related deaths. With the current treatment possibilities, a definitive, safe, and effective treatment approach for CRC has not been presented yet. However, new drug delivery systems show promise in this field. Amphiphilic cyclodextrin-based nanocarriers are innovative and interesting formulation approaches for targeting the colon through oral administration. In our previous studies, oral chemotherapy for colon tumors was aimed and promising results were obtained with formulation development studies, mucin interaction, mucus penetration, cytotoxicity, and permeability in 2D cell culture, and furthermore in vivo antitumoral and antimetastatic efficacy in early and late-stage colon cancer models and biodistribution after single dose oral administration. This study was carried out to further elucidate oral camptothecin (CPT)-loaded amphiphilic cyclodextrin nanoparticles for the local treatment of colorectal tumors in terms of their drug release behavior and efficacy in 3-dimensional tumor models to predict the in vivo efficacy of different nanocarriers. The main objective was to build a bridge between formulation development and in vitro phase and animal studies. In this context, CPT-loaded polycationic-β-cyclodextrin nanoparticles caused reduced cell viability in CT26 and HT29 colon carcinoma spheroid tumors of mice and human origin, respectively. In addition, the release profile, which is one of the critical quality parameters in new drug delivery systems, was investigated mathematically by release kinetic modeling for the first time. The overall findings indicated that the strategy of orally targeting anticancer drugs such as CPT with positively charged poly-β-CD-C6 nanoparticles to colon tumors for local and/or systemic efficacy is a promising approach.

## Introduction

Cancer is still one of the most common, highly variable and fatal diseases worldwide. Therefore, studies are continuing to develop effective/innovative and more flexible treatments for various types of cancer [1]. Colorectal cancers (CRC) are characterized by the presence of tumors that begin as polyps in the inner wall of the colon and rectum with uncontrolled growth. CRC is a common and metastatically aggressive disease ranking second in terms of cancer-related deaths [2]. Although surgical resection is possible, chemotherapeutic treatment is still one of the most researched approaches in terms of tumor recurrence and the progression of the disease. In CRC chemotherapy, the most common approach is mainly an intravenous administration of anticancer drugs such as camptothecin (CPT) analogs (irinotecan, topotecan), 5-fluorouracil (5-FU), oxaliplatin (OXA) or the combination of these drugs. However, current treatment approaches frequently result in major adverse effects, non-specific biodistribution, poor patient compliance, and clinically inadequate results in terms of efficacy [3–5]. Today, there is an intense focus on oral drug delivery, especially in the treatment of chronic diseases such as cancers. Even though there have been many developments in the field of chemotherapy in recent years, both in terms of diagnosis and treatment, oral chemotherapy has not yet been fully achieved due to the physicochemical properties and poor bioavailability of many widely used anticancer drugs.

Specifically in the treatment of CRC, since the colon is the most distant part of the gastrointestinal (GI) tract, the ability of oral delivery of anticancer drugs to reach the colon in a stable and effective structure is one of the biggest problems for researchers [6–9]. In the gastrointestinal environment, self-assembled nanoparticles are envisioned to protect the active ingredient from pH, enzymatic degradation, and efflux pumps in the intestines. Furthermore, the release profiles of the drug molecules from the dosage system may be controlled by altering the physicochemical parameters (particle size, zeta potential, hydrophobicity) of the self-assembling units utilized to design nanoparticles [9–12]. However, oral nano drug delivery systems capable of providing all the necessary features using polymeric nanoparticles for anticancer drug delivery have not been developed yet. Previous data showed encapsulation in non-ionic uncoated or coated cyclodextrin (CD) nanoparticles enhanced the CPT stability and GI absorption [9,11], and compared to PCL and PLGA nanoparticles, CD nanoparticles also had better release, physical stability, and cytotoxicity [13].

CPT is an anticancer small molecule drug that inhibits the topoisomerase I enzyme, which has a critical role in cellular DNA functions [14], and is effective in a wide spectrum of cancers such as metastatic colon cancer, breast cancer, and small cell lung cancer. It still has not been used clinically for CRC treatment due to its physiological instability and clinical inefficacy due to its physicochemical structure and hydrolytic degradation potential [9,13,15]. While the active lactone form of CPT is present at acidic pH, it is hydrolyzed to the ineffective carboxylate form at basic pH, resulting in decreased clinical efficacy and increased drug-related toxicity. As only the lactone structure of CPT can be transferred through cellular membranes and inhibit topoisomerase I, it is the functional component of CPT lactone form that is primarily responsible for the anticancer action [8,14,16–18]. To avoid CPT inactivation at alkaline medium, the concept that the lactone form can be kept stable by being encapsulated in an acidic microenvironment is also fascinating. Custom synthesized polycationic cyclodextrin amphiphiles have shown the ability to self-assemble into NPs amenable for encapsulation of an array of therapeutics (from small molecule drugs to nucleic acids) [19]. Moreover, their dense ammonium functional display furnishes their NPs with an overall positive charge and large buffering capabilities [19] at physiological pH. Preliminary studies have evidenced p*K*_a_ values in the 8.3 range for non-amphiphilic surrogates of these cationic CDs, supporting the cationic character of the resulting NPs [20]. We hypothesized that these polycationic CD NPs, which we designed in our previous studies, could protect the active and stable lactone form of encapsulated CPT due to their acidic chemical structure [8–9,11,13].

CDs are biocompatible cyclic polysaccharides formed by (1→4)-bound α-glucopyranose subunits obtained as a result of enzymatic degradation of starch by glucosyltransferase [21]. With their troncoconic structures having hydrophobic cavities and hydrophilic exterior surfaces, CDs are widely used in the pharmaceutical field to form inclusion complexes mostly with nonpolar molecules in their cavities [22]. In addition, CDs also offer several advantages for colonic drug delivery, because CDs are broken down by the intestinal microflora and dextrans are broken down by the endodextranases in the colon. Since they can predominantly be degraded by colonic microflora, CDs have been investigated in terms of drug delivery systems targeting the colon for many years [9,23–24]. Amphiphilic CD nanocarriers have been extensively investigated in new drug and gene delivery studies, particularly in cancer therapy, for targeted drug delivery, extended/controlled release, and improving cellular interaction [25–29].

Within the scope of this study, advanced studies were carried out for the oral polycationic nanodrug delivery system, developed in our previous research for the treatment of CRC to build a bridge between in vitro characterization and in vivo animal efficacy studies and to establish a screening tool for nanoparticulate formulations for poorly bioavailable anticancer drugs administered through a non-parenteral route.

In this context, release kinetic modeling studies and 3D cell culture studies of colon carcinoma cells of mice and human origin were carried out for the first time for CPT-loaded positively charged β-CD nanoparticles with different formulations. A positive surface charge was achieved through either (i) the cationic nature of the CD such as poly-β-CD-C6 or (ii) coating of non-ionic 6-O-capro-β-CD with the cationic polymer chitosan (CS). Uncoated 6-O-capro-β-CD negatively charged NPs were used as control formulation.

## Results and Discussion

### Fabrication and in vitro characterization of CPT-loaded amphiphilic CD NPs

CPT-loaded amphiphilic CD nanoparticles have been previously optimized in our laboratories [9], as reported, NPs using two different amphiphilic CDs were prepared and 6-O-capro-β-CD nanoparticles coated with chitosan (CS) to obtain a positively charged surface. In vitro characterization and cell culture studies for 6-O-capro-β-CD, CS-(6-O-capro-β-CD), and poly-β-CD-C6 formulations have been comprehensively evaluated previously [9]. According to the pre-formulation studies, an optimal formulation with desired characteristics was determined as CPT/poly-β-CD-C6 NPs with a 135 nm particle size, very low polydispersity index, and a zeta potential of + 40 mV. In vitro release experiments showed that amphiphilic CD NPs have properties suitable for colon targeting, but the most promising were poly-β-CD-C6 NPs with 52% of encapsulated CPT successfully delivered all the way to the simulated colon. When compared to the equivalent CPT dose in solution, CPT-loaded poly-β-CD-C6 nanoparticles exhibited higher cytotoxicity in HT-29 cells. Permeability studies performed with the Caco-2 cell line revealed a 276% increase in drug permeability and significantly higher intestinal penetration with the cationic CD formulation. In our further research [8], it was also reported that the oral CPT-loaded poly-β-CD-C6 NPs showed antitumoral and antimetastatic effects in a colorectal tumor-bearing animal model.

### Drug release from amphiphilic CD nanoparticles

**Figure 1:**
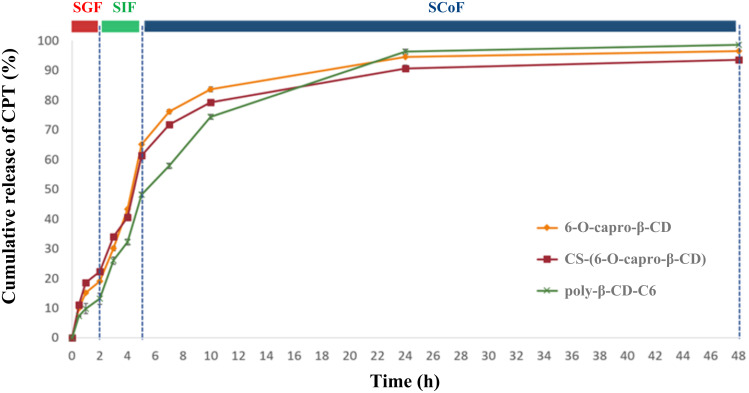
In vitro release profile of CPT from nanoparticle formulations (*n* = 3, ± SD).

In vitro release studies were performed over 48 hours in order to clearly elucidate the release kinetics (Figure 1). An in vitro release study was carried out at 0–2 hours in simulated gastric fluid (SGF), 2–5 hours in simulated intestinal fluid (SIF), then in simulated colonic fluid (SCoF) settings till the completion of the experiment in order to imitate GIT circumstances in terms of pH and transit duration. The purpose of the release study was to elucidate the ability of the formulation to retain the encapsulated drug in the stomach and small intestine and preferably release it when it reaches the colon. The optimum nanoparticle formulation was considered to deliver most of the effective lactone-form CPT to the colon.

It is known that it takes approximately 5 hours for oral drug delivery systems to reach the colon; the first 2 hours in the stomach and the last 3 hours in the small intestine [30]. At the end of the 5th hour, 6-O-capro-β-CD and CS-(6-O-capro-β-CD) formulations revealed faster release profiles (*p* > 0.05) than poly-β-CD-C6 nanoparticles. Poly-β-CD-C6 nanoparticles showed a slower release of CPT (48%) until the colonic area as compared to the other formulations (*p* < 0.05). Previous research has looked into detailed assessments of this topic [9].

### Release kinetics study

**Figure 2:**
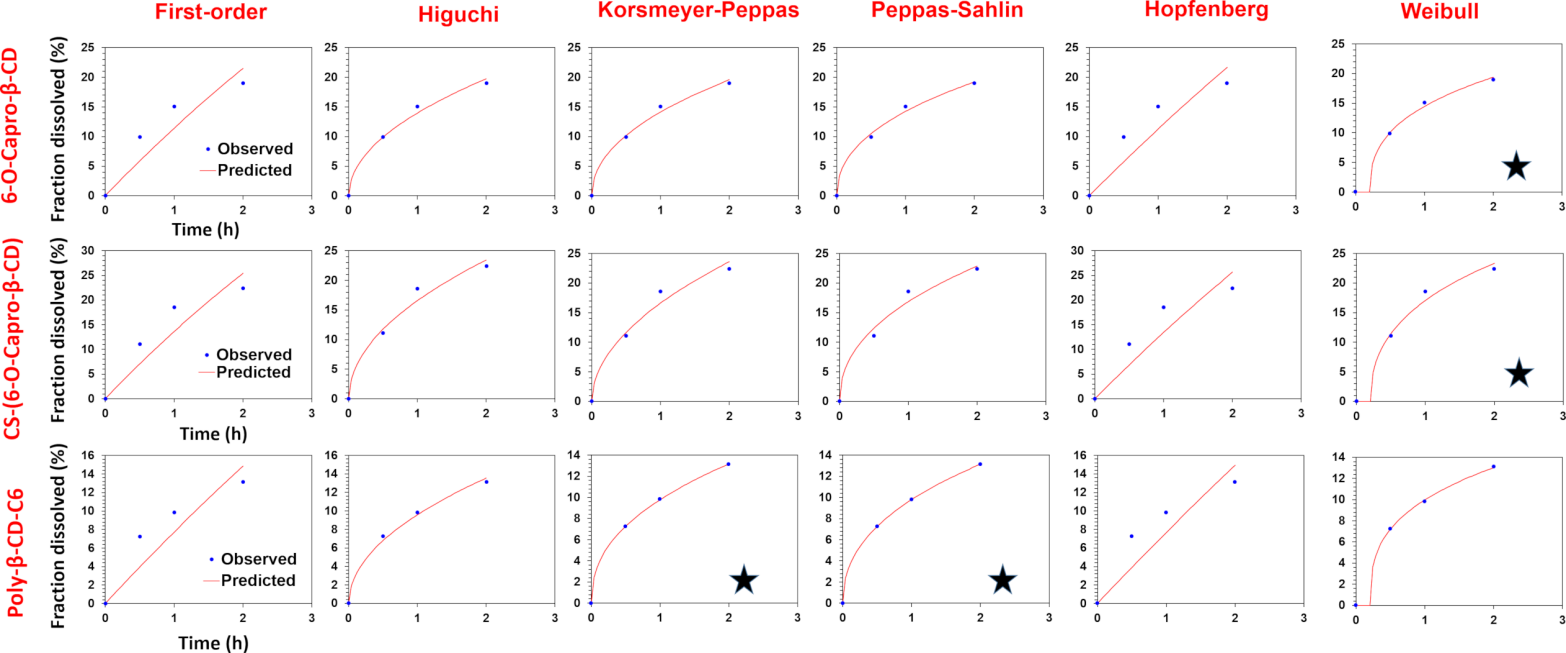
Results for release kinetics obtained automatically by the DDSolver software for SGF release medium (*represents best fit models).

**Figure 3:**
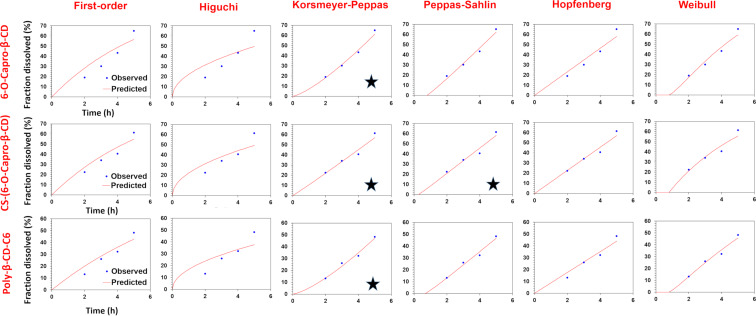
Results for release kinetics obtained automatically by the DDSolver software for SIF release medium (*represents best fit models).

**Figure 4:**
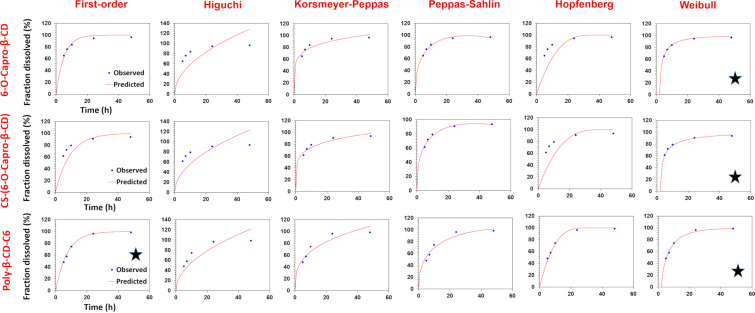
Results for release kinetics obtained automatically by the DDSolver software for SCoF release medium (*represents best fit models).

The in vitro release profiles of CPT-loaded amphiphilic cyclodextrin nanoparticles were fitted with a variety of kinetic models, and the release mechanisms, which are illuminating markers for novel drug delivery systems, were mathematically investigated. In this context, 6 models (first order, Hopfenberg, Korsmeyer–Peppas, Higuchi, Peppas–Sahlin, and Weibull models) and 3 criteria (coefficient of determination (R^2^), Akaike information criterion (AIC) and model selection criterion (MSC)) were evaluated for the in vitro release profiles. Much of the research in this field generally evaluates the kinetic data of the total release profiles of the nanoparticles, although it is useful to look at potential alterations in the release kinetics at different release mediums (SGF, SIF, SCoF) as well, especially in orally administered drug delivery systems. To achieve this, a thorough and in-depth release kinetic study was conducted, and the parameters were compared for the GIT conditions. Table 1 displays the findings of the release kinetic modeling studies and graphical reports are presented in Figure 2, Figure 3, and Figure 4. Figures 2–4 show that the kinetic models' predicted and observed CPT releases appear to be consistent with formulations for the best correlated models. Thus, the mathematical compatibility of the kinetic models' graphics with good correlation was also proven. Furthermore, as seen in Table 2, the release profiles of CPT from different formulations were compared in terms of similarity (*f*2) and difference (*f*1) factors, and the results revealed that the release profiles of nanoparticles, which we obtained using the same formulation parameters with structurally similar amphiphilic cyclodextrin derivatives, showed similar release profiles. Formulation parameters affected the release kinetics of the drug-loaded nanoparticles [31–32].

**Table 1:** Release kinetic modelling and results of NP formulations.

Model and equation	SGF				SIF				SCoF			

	0–2 hours kinetics				2–5 hours kinetics				5–48 hours kinetics			
	R^2^	AIC	MSC	*n*/m*	R^2^	AIC	MSC	*n*/m*	R^2^	AIC	MSC	*n*/m*

6-O-Capro-β-CD												

First-order F = 100·[1−Exp(−k1·t)]	0.823	16.307	0.328	–	0.765	24.496	0.947	–	0.929	21.434	2.243	–
Higuchi F = kH·t^0.5	0.991	4.366	3.313	–	0.606	26.554	0.432	–	−3.300	41.940	−1.859	–
Korsmeyer–Peppas F = kKP·t^n	0.993	5.205	3.103	0.471	**0.982**	**16.301**	**2.996**	**1.319**	0.861	26.790	1.171	0.163
Peppas–Sahlin F = k1·t^m + k2·t^(2·m)	0.994	6.564	2.764	0.450	0.976	19.342	2.235	0.450	0.975	20.277	2.474	0.450
Hopfenberg F = 100·[1−(1−kHB·t)^n]	0.809	18.610	−0.248	3.000	0.915	22.416	1.467	1.000	−2.188	42.443	−1.959	3.000
Weibull F = 100·{1−Exp[−((t−Ti)^β)/α]}	**0.997**	**3.640**	**3.495**	**–**	0.946	22.605	1.420	–	**0.987**	**17.087**	**3.112**	**–**

CS-(6-O-Capro-β-CD)												

First-order F = 100·[1−Exp(−k1·t)]	0.830	17.597	0.406	–	0.846	21.276	1.368	–	−0.370	36.415	−0.715	–
Higuchi F = kH·t^0.5	0.981	11.809	2.603	–	0.685	24.130	0.654	–	−2.498	41.103	−1.652	–
Korsmeyer–Peppas F = kKP·t^n	0.980	10.967	2.064	0.507	**0.949**	**18.874**	**1.968**	**1.039**	0.886	25.998	1.369	0.176
Peppas–Sahlin F = k1·t^m + k2·t^(2·m)	0.983	12.359	1.716	0.450	**0.950**	**20.802**	**1.486**	**0.450**	0.983	18.406	2.887	0.422
Hopfenberg F = 100·[1−(1−kHB·t)^n]	0.814	19.964	−0.185	3.000	0.944	19.206	1.885	1.000	−2.105	42.508	−1.933	3.000

Poly-β-CD-C6												

First-order F = 100·[1−Exp(−k1·t)]	0.805	13.614	0.192	–	0.847	20.320	1.377	–	**0.992**	**16.096**	**4.389**	**–**
Higuchi F = kH·t^0.5	0.995	−1.199	3.895	–	0.627	23.886	0.485	–	0.404	37.459	0.117	–
Korsmeyer–Peppas F = kKP·t^n	**0.999**	**−18.653**	**8.259**	**0.429**	**0.978**	**14.572**	**2.814**	**1.362**	0.862	32.156	1.177	0.318
Peppas–Sahlin F = k1·t^m + k2·t^(2·m)	**0.999**	**−20.534**	**8.729**	**0.450**	0.975	17.011	2.204	0.450	0.935	30.393	1.530	0.450
Hopfenberg F = 100·[1−(1−kHB·t)^n]	0.795	15.806	−0.356		0.927	19.383	0.952	1.000	0.985	21.122	3.384	3.188
Weibull F = 100·{1−Exp[−((t−Ti)^β)/α]}	0.996	−7.005	5.346	**–**	0.967	18.149	1.920	–	**0.991**	**20.463**	**3.516**	**–**

**Table 2:** Difference and similarity factors between formulations.

CPT-loaded amphiphilic CD nanoparticles		difference factor *(f*1)	similarity factor *(f*2)

6-O-capro-β-CD	CS-(6-O-capro-β-CD)	6.63	73.82
CS-(6-O-capro-β-CD)	poly-β-CD-C6	13.73	58.86
poly-β-CD-C6	6-O-capro-β-CD	11.72	61.57

According to the release kinetic parameters in SGF medium, as seen in Table 1, the highest R^2^, MSC and lowest AIC values were observed in the Weibull model for 6-O-capro-β-CD and CS-(6-O-capro-β-CD) formulations, and in the Korsmeyer–Peppas and Peppas–Sahlin models for the poly-β-CD-C6 formulation. For the poly-β-CD-C6 NPs, two models were found to be compatible with high correlation. There are also studies in the literature indicating that the release kinetic model of nanoparticles can fit to more than one model [33–34]. Since it is the first study to evaluate the release kinetics of amphiphilic cyclodextrin nanoparticles, we have reported that a drug release profile that fits more than one model can be observed for amphiphilic cyclodextrin nanoparticles. In the Weibull model, the “β” (shape parameter of the release curve) value is a criterion used to illuminate the release from a polymeric matrix. “β” ≤ 0.75 indicates Fickian diffusion, while 0.75 < “β” < 1 indicates Fickian diffusion and controlled release combination [35]. The “β” value for the Weibull model was calculated as 0.396 and 0.434 for the 6-O-capro-β-CD and CS-(6-O-capro-β-CD) nanoparticle formulations, respectively. According to the Weibull model, CPT release kinetics from nanoparticles were found to be compatible with Fickian diffusion in SGF medium [36]. In the model-independent principal evaluation of in vitro release profiles, this was considered as the rapid/burst and initial release of the drug adsorbed on the nanoparticle surface or encapsulated in the nanoparticle material matrix. It has been confirmed by mathematical modeling that the release is based on diffusion. This indicates the release of the CPT, which is weakly bound in the nanoparticle matrix and adsorbed on the surface, for the 6-O-capro-β-CD and CS-(6-O-capro-β-CD) formulations. These results we obtained confirm each other with the data we interpreted in our previous studies [9]. The release kinetics, however, also appeared to be consistent with the Korsmeyer–Peppas and Peppas–Sahlin models for poly-β-CD-C6 NPs. While the Korsmeyer–Peppas model expresses diffusion-controlled release from matrix-type nanosystems, the Peppas–Sahlin model is based on the combination of diffusion and erosion of the nanoparticle matrix. In order to further elucidate the kinetics of these models, the diffusional exponent values (*n* or *m*) regarding the release kinetics from the nanoparticles were computed [37]. In the Korsmeyer–Peppas model, "*n*" represents the diffusional exponent illustrating the drug release mechanism, but in the Peppas–Sahlin model, "*m*" represents the same parameter [38]. In this context, "*m*" and "*n*" diffusional exponent values were computed as 0.450 and 0.429, respectively. A diffusional exponent (*m*/*n*) ≤ 0.45 indicates that Fickian diffusion is a factor in drug release [39]. For 0.45 < *m*/*n* < 0.85, the drug release occurs through a non-Fickian diffusion mechanism, for *m*/*n* = 0.85 the release occurs by case II transport and *m*/*n* > 0.85 indicates super case II transport [38–42]. It has been determined that there is Fickian diffusion in the release kinetics based on the diffusional exponent values of the Korsmeyer–Peppas and Peppas–Sahlin models [39]. When all the data were analyzed together, it was determined that a single kinetic model was not dominant in the SGF release kinetics, and compliance with different models was observed. However, although the models were different in all formulations, it was observed that the dominant mechanism was diffusion-based release. In our previous studies, it was evaluated that the drug release observed in SGF might be related to the diffusional release of the surface-adsorbed drug and the poorly bound surface drug to the matrix. In the mathematical modeling data, it has been confirmed that the first release seen in the SGF medium is dominantly diffusion-related.

According to the release kinetic parameters in SIF medium, as seen in Table 1, the highest R^2^, MSC and lowest AIC values were observed in the Korsmeyer–Peppas model for 6-O-capro-β-CD and poly-β-CD-C6 NPs, and in Korsmeyer–Peppas and Peppas–Sahlin models for CS-(6-O-capro-β-CD) formulation. Two models were found to be compatible with high correlation for the CS-(6-O-capro-β-CD) formulation for SIF medium. Similarly, studies showing that nanoparticles can fit more than one model in the literature were also mentioned for SGF data [43]. The Korsmeyer–Peppas model difussional exponent values (*n*) for 6-O-capro-β-CD and poly-β-CD-C6 NPs were computed as 1.319 and 1.362, respectively. Considering the difussional exponent data over 0.85 indicates that the release mechanism is compatible with super case II transport. Case II transport refers to the release that occurs as a result of relaxation of the polymeric structure [40,42]. These results were interpreted as supporting our idea that the release of the drug adsorbed to the surface is completed by diffusion in the SGF medium, and that the erosion of the nanoparticle material and the relaxation of the polymer chain begins and accelerates the release in SIF. When a further evaluation was made for the CS-(6-O-capro-β-CD), which showed a high correlation between the two models, the diffusional exponent values of the Korsmeyer–Peppas and Peppas–Sahlin models were calculated as 1.039 and 0.450, respectively. Similarly, the *n* value of Korsmeyer–Peppas above 0.85 indicates that the release mechanism is realized by super case II [44]. This value was interpreted as indicative of the release seen with the erosion of the nanoparticle material and the initiation of polymer relaxation [45]. On the other hand, the “*m*” value calculated as 0.45 in the Peppas–Sahlin model indicates Fickian diffusion. This situation was evaluated as a very significant and meaningful data when compared with other formulations. Unlike the other two formulations, CS-(6-O-capro-β-CD) is coated on its surface with chitosan, a cationic coating material. The theoretical interpretations so far have been that the drug can be adsorbed in the coating material or weakly bound to the coating polymer structure, and it will be released first. The data obtained from the kinetic modeling provided results that support this interpretation. For the 6-O-capro-β-CD and poly-β-CD-C6 NPs (uncoated formulations), it was confirmed that the release occurred as a result of the relaxation of the nanoparticle material in SIF, while the Fickian diffusion continued for the CS-(6-O-capro-β-CD) formulation, that is, the release of the weakly bound drug adsorbed on the coating material. The diffusional exponent values in SIF for CS-(6-O-capro-β-CD) showed that the release continues as a combination of both the diffusion release of the drug adsorbed to the coating material, chitosan, and the case II release, which occurs as a result of the relaxation of the nanoparticle polymer structure [46].

According to the release kinetic parameters in the targeted main release medium, SCof, the highest R^2^, MSC, and lowest AIC values were observed in the Weibull model for 6-O-capro-β-CD and CS-(6-O-capro-β-CD) formulations, and in the first order release and Weibull models for the poly-β-CD-C6 NPs, as seen in Table 1. In the Weibull model, the “β” (shape parameter of the release curve) exponent is a parameter used to elucidate the release from a nanoparticle matrix. “β” ≤ 0.75 indicates Fickian diffusion, while 0.75 < “β” < 1 indicates a complex mechanism (Fickian diffusion and controlled release). For values of “β” higher than 1, it was demonstrated that the drug transport follows a complex release mechanism [35,47–48]. The “β” value for the Weibull model was calculated as 0.493 and 0.401 for the 6-O-capro-β-CD and CS-(6-O-capro-β-CD) NPs, respectively. When evaluated within the framework of the literature, it was determined that the release mechanism of encapsulated CPT in SCof medium is by Fickian diffusion [36]. In addition, this situation has also been interpreted as further relaxation of the nanoparticle matrix structure in the SCoF medium, making diffusion easier and coming to the fore as a primary release mechanism [49]. On the other hand, the other formulation, the poly-β-CD-C6 NPs, was found in accordance with both Weibull and first order kinetics. According to the Weibull model, the “β” value was calculated as 0.762. Within the framework of the information explained above, it was evaluated as a complex (Fickian diffusion and controlled release) release mechanism according to the Weibull model for the poly-β-CD-C6 NPs. As stated in the literature, values of “β” in the range of 0.75–1.0 indicate a combined mechanism which is frequently encountered in release studies. When the power law can adequately represent the whole collection of data in these situations, further confirmation can be gained. The special case of “β” = 1 is compatible with first order release, whereas the concentration gradient in the dissolution medium drives the rate of release [35]. In our calculations, results compatible with first order kinetics were found for the poly-β-CD-C6 and it was considered to fit both models. The partially high “β” value for the Weibull model also confirmed the tendency towards first order kinetics, which is also evaluated in the previous sentence in line with the literature. In this context, it has been evaluated that the first order kinetics associated with diffusion in the SCoF medium for the poly-β-CD-C6 formulation also occurs as a release mechanism. It was observed that the Weibull and first order models were compatible, supported and confirmed each other, providing an explanatory idea about CPT release from the formulation.

### Cell culture studies

#### Determination of IC_50_ values of camptothecin

CT26 and HT29 cells were incubated with increasing concentrations of CPT and different CD nanoparticle formulations containing equal amounts of camptothecin for 48 or 72 hours. When the incubation period was over (48 or 72 h), cell viability was determined with the WST-1 assay. IC_50_ values are shown in Table 3.

**Table 3:** IC_50_ (µM) values of CPT solution and CPT-loaded CD nanoparticle formulations for CT26 murine and HT29 human colon cancer cell lines at 48 h and 72 h (*n* = 6, mean ± SD).

Formulation	CT26		HT29	
	48 h	72 h	48 h	72 h

CPT/6-O-capro-β-CD	1.23 ± 0.02	1.19 ± 0.06	0.76 ± 0.09	0.58 ± 0.14
CPT/CS-(6-O-capro-β-CD)	0.72 ± 0.26	0.59 ± 0.12	0.89 ± 0.07	0.59 ± 0.16
CPT/poly-β-CD-C6	1.35 ± 0.46	0.61 ± 0.14	0.30 ± 0.03	0.25 ± 0.04
CPT solution in DMSO	1.86 ± 0.28	1.27 ± 0.42	1.47 ± 0.06	1.31 ± 0.06

It was observed that the IC_50_ values of the drug solution and nanoparticle formulations in each cell line were different and the IC_50_ values of CPT-loaded nanoparticles was lower than the CPT solution in both cell lines. For CT26 cells, the IC_50_ values of the drug solution was calculated as 1.86 ± 0.28 µM and 1.27 ± 0.42 µM for 48 h and 72 h, respectively. Among different formulations, the CS-(6-O-capro-β-CD) nanoparticle formulation had the highest efficiency for both time points against the CT26 cell line. After 48 hours of incubation, the IC_50_ values of anionic CPT-loaded 6-O-capro-β-CD and CPT-loaded poly-β-CD-C nanoparticles were calculated as 1.23 ± 0.02 µM and 1.35 ± 0.46 µM, respectively, and the difference between the two groups was not statistically significant (*p* > 0.05). However, after 72 hours of incubation, the IC_50_ value of the anionic nanoparticles was found to be two folds of the cationic nanoparticles. Considering these findings, it is thought that the difference between the IC_50_ values of three different nanoparticle formulations containing equal amounts of drug may be related to the surface charges of the nanoparticles. For CT26 cells, when 6-O-capro-β-CD and CS-(6-O-capro-β-CD) nanoparticle formulations are compared, it can be interpreted that the switch of NP charge upon chitosan coating enhances this membrane binding ability. However, there was no significant difference in IC_50_ values against HT29 cells between 6-O-capro-β-CD and CS-(6-O-capro-β-CD) groups. Due to the increased biological membrane interaction, the amount of drug transported into the cell may also have increased. The 6-O-capro-β-CD and poly-β-CD-C6 derivatives used in the study are cyclodextrin derivatives with the same core structure. Heptakis(6-*O*-hexanoyl)-β-CD (6-O-capro-β-CD) is a primary face-modified amphiphilic CD derivative with a 6C fatty acid chain attached via an ester bond to the primary hydroxy groups of the macrocyclic ring. On the contrary, poly-β-CD-C6 is furnished with a set of primary aminoethyl segments on the primary rim of the β-CD core and a cluster of 14 hexanoyl chains on the secondary face. The effect of the change in surface modifications on cell viability has been demonstrated by cell culture studies performed within the scope of this paper. The anionic 6-O-capro-β-CD nanoparticle formulation has a lower IC_50_ value after 48 hours than the polycationic poly-β-CD-C6 nanoparticle formulation. However, it was observed that the IC_50_ value of the poly-β-CD-C6 nanoparticle formulation decreased upon incubation. Based on previous studies with breast cancer cell lines, it is comprehensible that anionic nanoparticles induce cell proliferation inhibition earlier than polycationic nanoparticles. The impact of anionic nanoparticles on free cholesterol level was shown to decrease after 24 hours in a cholesterol extraction assay from MCF-7 cells. Poly-β-CD-C6 nanoparticles, on the other hand, removed three times more cholesterol from cells in 48 hours than anionic CD nanoparticles. In addition to surface charges, the molecular weight, and number of aliphatic groups on the surfaces of CD derivatives play a direct role in the interaction time with the cell and cell membrane components [50]. It is well established that positively charged nanoparticles interact with the cell membrane more favorably than negatively charged ones. However, their passing through the cell membrane is challenging because of the agglomeration of positive charge on the cell membrane [51]. This knowledge might explain why the 6-O-capro-β-CD nanoparticle IC_50_ values are lower at 48 hours than those of the poly-β-CD-C6 nanoparticles. Besides, chitosan coating on nanoparticles reduced the IC_50_ value. It is believed that chitosan's antiproliferative properties also are involved in this situation.

When cell viability in HT29 cells was evaluated, it was observed that the polycationic derivative had the lowest IC_50_ value. It can be said that the chitosan coating did not cause a positive change in 6-O-capro-β-CD nanoparticles. It is known that the same nanoparticles may have different effects on cancer cells of different species. In addition to the surface charges, particle size and distribution also play a very important role in the cellular interactions of nanoparticles. For this reason, while evaluating the effects of nanoparticles, the selection of particles with the most ideal parameters for the target disease or organ is very important in terms of the effectiveness of the treatment. Due to the differences between colorectal cancer cells of different species, it is possible that the same formulations have different effects on these cells, and this result supports the publications in the literature [52–54].

#### Determination of doubling time

After 48 hours, 42.410 cells/mL for CT26 and 37.112 cells/mL for HT29 were counted. According to Equation 1, doubling times were calculated as 23.03 h and 25.37 h, respectively.

The factors affecting cells in 2D and 3D cell culture media are quite different from each other. In addition to physiological differences, cell culture protocols are also different. In conventional 2D cell culture studies, cells proliferate and then are detached with trypsin and inoculated on appropriate plates followed by overnight incubation for attachment. In the 3D cell culture method, cells are plated after detachment and allowed to form a spheroid for more than 24 hours (i.e., 3 days for this study). Due to the differences in the initial incubation time between the two methods, the number of cells treated with nanoparticles at the beginning of the experiment is quite different. Therefore, to equalize the number of cells between the two methods, the doubling times of the cells were calculated as detailed in the methods section.

#### Evaluation of anticancer efficiency of CPT-loaded nanoparticles on 2D cell cultures

**Figure 5:**
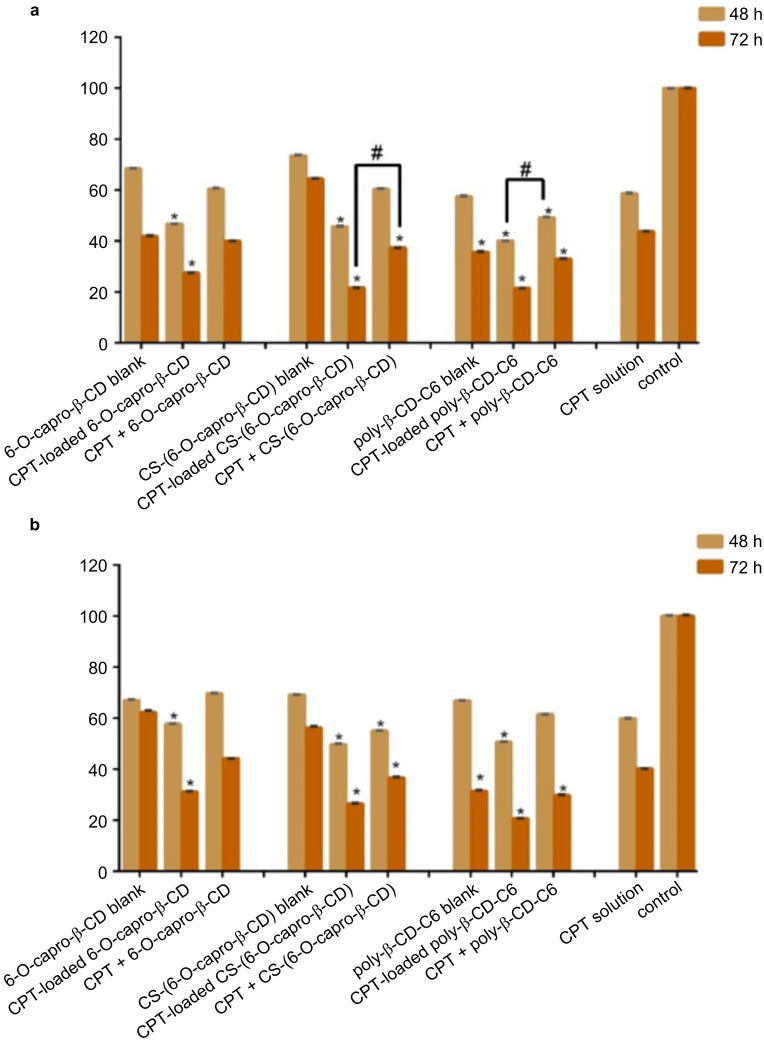
Anticancer effect of blank or CPT-loaded CD nanoparticles and camptothecin solution against 2D CT26 murine (a) and HT29 human (b) colon cancer cell lines at 48 h and 72 h. Cell viability was evaluated by WST-1 assay. (*n* = 6, mean ± SD), (^#^ *p* < 0.05 and * *p* < 0.05 compared with CPT solution in DMSO).

According to the results of the conventional 2D cell culture study, the CPT-loaded poly-β-CD-C6 nanoparticle treatment group in CT26 cells had the highest antiproliferative effect after 48 h (Figure 5a). When compared to CPT solution-treated cells, cell viability was considerably reduced in the CPT-loaded poly-β-CD-C6 nanoparticle formulation and the CPT solution + blank poly-β-CD-C6 nanoparticle formulation groups at the conclusion of the 48-hour incubation period. For 6-O-capro-β-CD and CS-(6-O-capro-β-CD) nanoparticles, the co-administration of blank nanoparticles and drug solution was shown to be more efficient in terms of cell survival than the drug solution alone at the end of the 72-hour incubation. The efficiency of drug-loaded nanoparticles is higher than that of co-administered formulations in all three nanoparticle dispersions (Figure 5a). Considering that the amount of drug and carrier is equal, it is thought that loading the drug into the nanoparticles may have increased cellular uptake and the amount of accumulated drug.

When data from a 48-hour anticancer efficiency on HT29 cells was evaluated, it was observed that the drug-loaded nanoparticle formulations had better anticancer activity than the drug solution (Figure 5b). Moreover, the group treated with blank CS-(6-O-capro-β-CD) nanoparticles + CPT solution showed a substantial reduction in cell viability. Cell viability in HT29 cells cultured with CPT solution alone was calculated to be 40% after 72 hours of incubation (Figure 5b). Cell viability of drug-loaded nanoparticles was determined as 20.6%, 26.5%, and 31.2% for 6-O-capro-β-CD, CS-(6-O-capro-β-CD), and poly-β-CD-C6 nanoparticles, respectively. In addition to the chitosan-coated nanoparticles, it was determined that the blank poly-β-CD-C6 nanoparticles plus CPT solution treated group had higher anticancer activity than the only drug solution treated group at the end of 72 hours. Furthermore, the anticancer activity of blank poly-β-CD-C6 nanoparticles was shown to be greater than that of the drug solution. It was established that both drug-loaded nanoparticles and drug + nanoparticles demonstrated better effectiveness than the drug solution in 72-hour incubation results in CT26 cells. The CPT-loaded CS-coated nanoparticle and poly-β-CD-C6 nanoparticle formulations, on the other hand, had the lowest cell viability.

**Figure 6:**
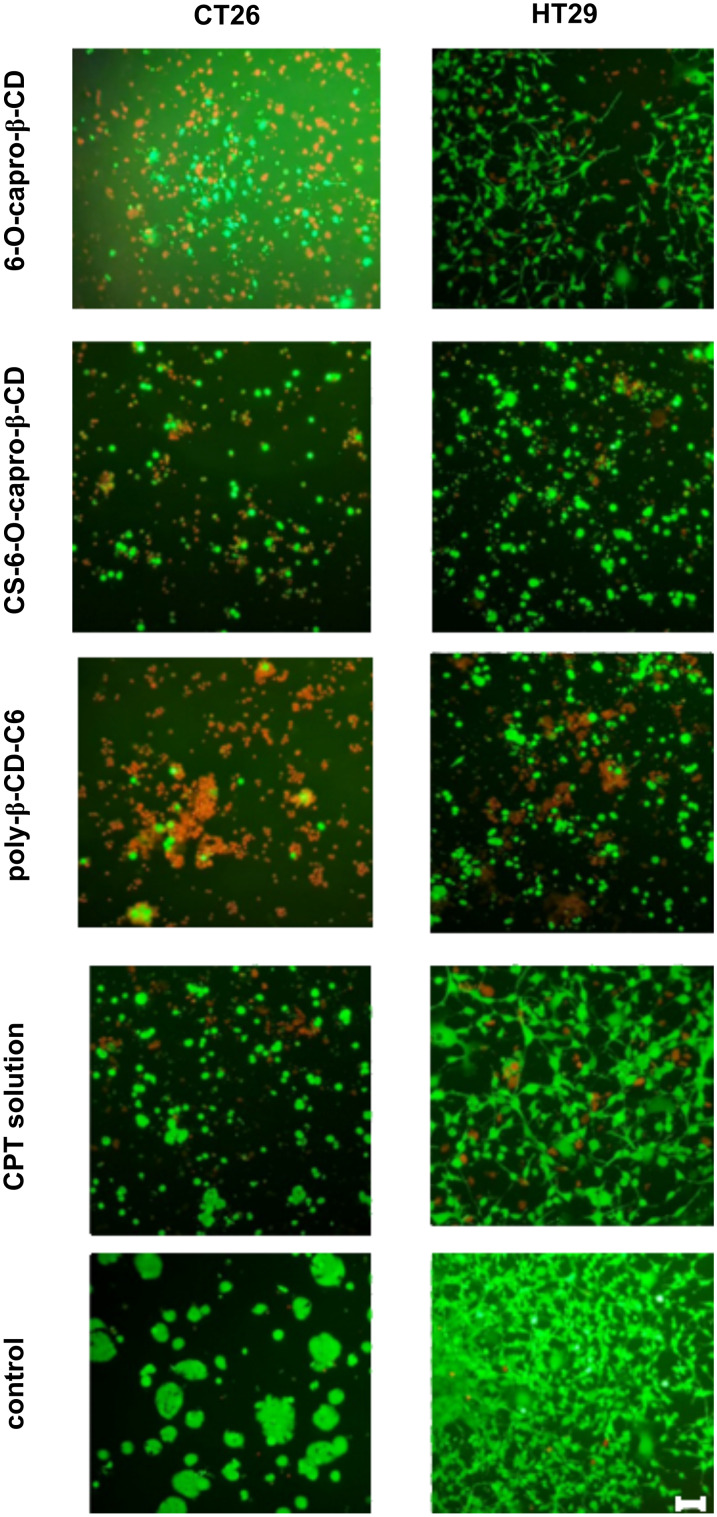
Live/dead analysis of CT26 and HT29 cells using double staining with calcein AM and ethidium homodimer-1 (EthD-1) after treatment with different formulations at 48 h. Control group is treated with only complete Dulbecco’s modified Eagle medium (DMEM). Live cells stained with calcein AM fluoresce green while dead cells stained with EthD-1 fluoresce red. Scale bar: 100 µm.

According to the results of the study performed with colon cancer cell lines of two different origin (murine and human), it was observed that the blank nanoparticles caused a decrease in cell viability (to <70%). Similar results were obtained in cell culture studies performed on different cancer cells by our group, and detailed studies were carried out to elucidate the mechanism. Both the results of our studies and the literature emphasize that cyclodextrins show high affinity for lipid-based molecules such as cholesterol and phospholipids in biological membranes [50,55–56]. Furthermore, it was reported that depletion of cholesterol by methyl-β-cyclodextrin could inhibit EGFR signals, induce apoptosis, and suppress tumor growth in colon tumor-induced mice [57]. Shimolina et al. evaluated membrane fluidity in CT26 and HeLa Kyoto cells treated with cisplatin in a monolayer conventional cell culture. It was emphasized that the increased plasma membrane fluidity due to the decrease of lipid rafts and, moreover, cholesterol in the biological membrane plays a role in inducing apoptosis [58]. When the literature information and the findings are evaluated, it can be said that the use of CD nanoparticles in the treatment of colon cancer can make it possible to reduce the amount of anticancer drugs required for treatment by taking advantage of the synergistic effect. The morphological change in CT26 and HT29 cells treated with different nanoparticle formulations was also examined microscopically. As seen in Figure 6, cells were double-stained with calcein AM and ethidium homodimer-1 (EthD-1). The control group consisted of cells incubated only with the medium. Living cells were stained green with the membrane dye calcein AM, while dead cells were stained red with the nuclear dye EthD-1. Both the decrease in cell number and the change in cell morphology draw attention in the microscopic images. In particular, in the CT26 cell line incubated with poly-β-CD-C6 nanoparticles the presence of red-labeled dead cells was observed. Especially in HT29 cells, it is noteworthy that the cellular interaction in the control group was not observed in the groups treated with nanoparticles. Similarly, it was determined that incubation with nanoparticles caused a change in the colonization of CT26 cells. Based on the results of both mitochondrial functional activity and microscopic imaging analyses following staining, CT26 cells are more sensitive to formulations than HT29 cells. The underlying processes must be elucidated in order to explain this variation. In fact, this is an expected result considering the origins of the cells. The primary factor causing the effects of nanoparticle formulations on two different colon tumors to differ from each other is the origin of the cells. The genome-transcriptome mapping investigation revealed that despite having two separate origins, the CT26 cell has characteristics similar to human primary colorectal cancers in terms of drug resistance mechanisms, gene expression and mutation patterns, and pathways in onco-related genes [59–60]. Nevertheless, because of their different origins, these cell lines exhibit various genetic and epigenetic changes as well as mutations due to their diverse origins. Efficacy/cytotoxicity studies on both cell lines indicate that cells respond differently to the treated groups [61–63].

#### Evaluation of antitumoral efficiency of CPT-loaded CD nanoparticles on 3D cell cultures

Anticancer activity of nanoparticles prepared from different CD derivatives was also investigated in a 3D cell culture method. Matrigel^®^ was used as the extracellular matrix in 3D cultures of colorectal cancer cell lines prepared using polymer-based scaffolds.

**Figure 7:**
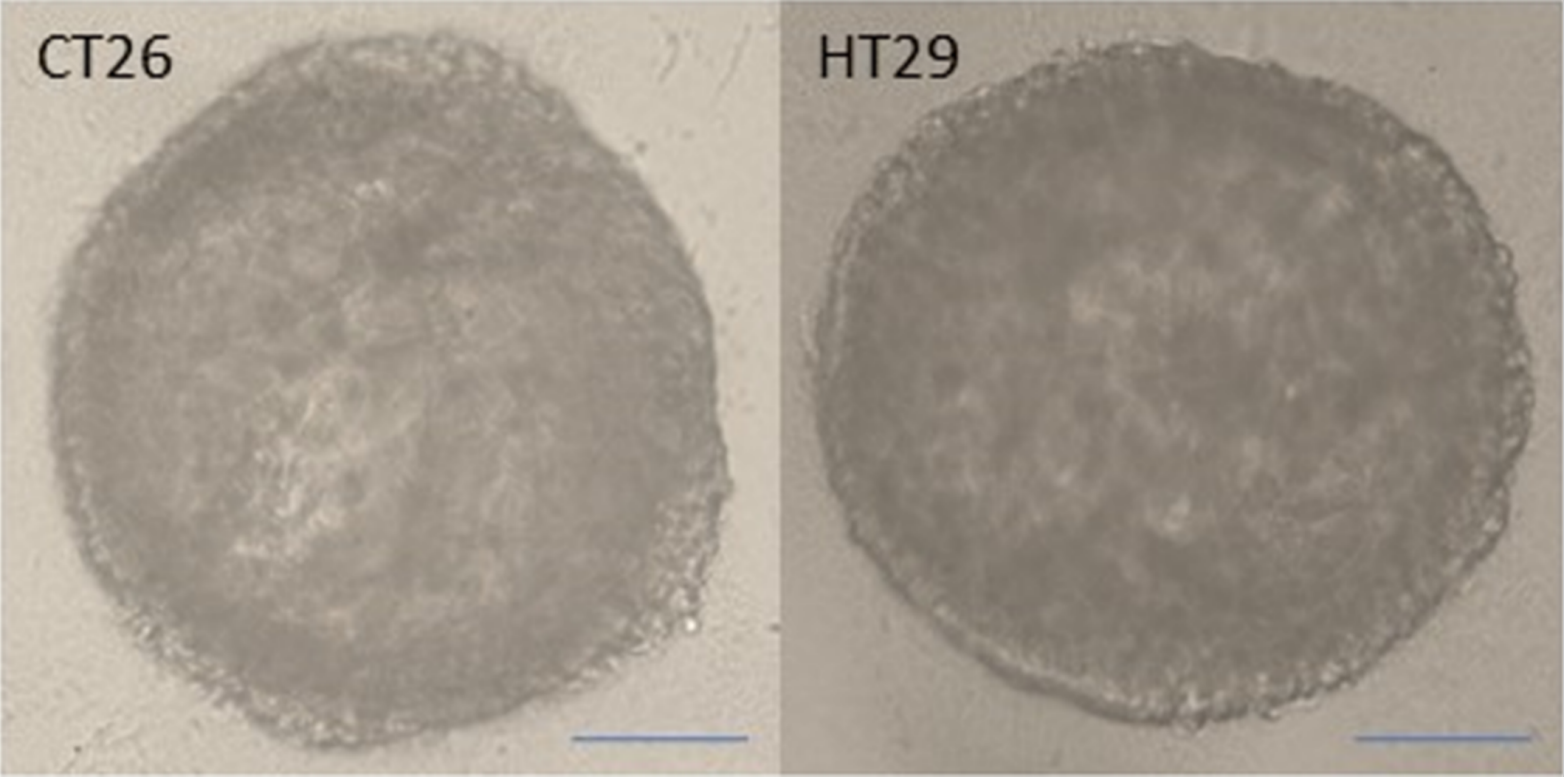
Murine (CT26) and human (HT29) colon cancer spheroid was formed by scaffold-based method, and the morphological structure was observed under a light microscope. Scale bar: 50 µm.

Murine or human colon cancer cells were seeded on poly-HEMA-coated cell plates prepared as described in the methods section, and the plates were centrifuged. The cells were found to be collected in the middle of the wells after centrifugation. Within 3 days, cells that interacted maximally with each other formed highly spherical tumors measuring about 200 μm in diameter (Figure 7).

**Figure 8:**
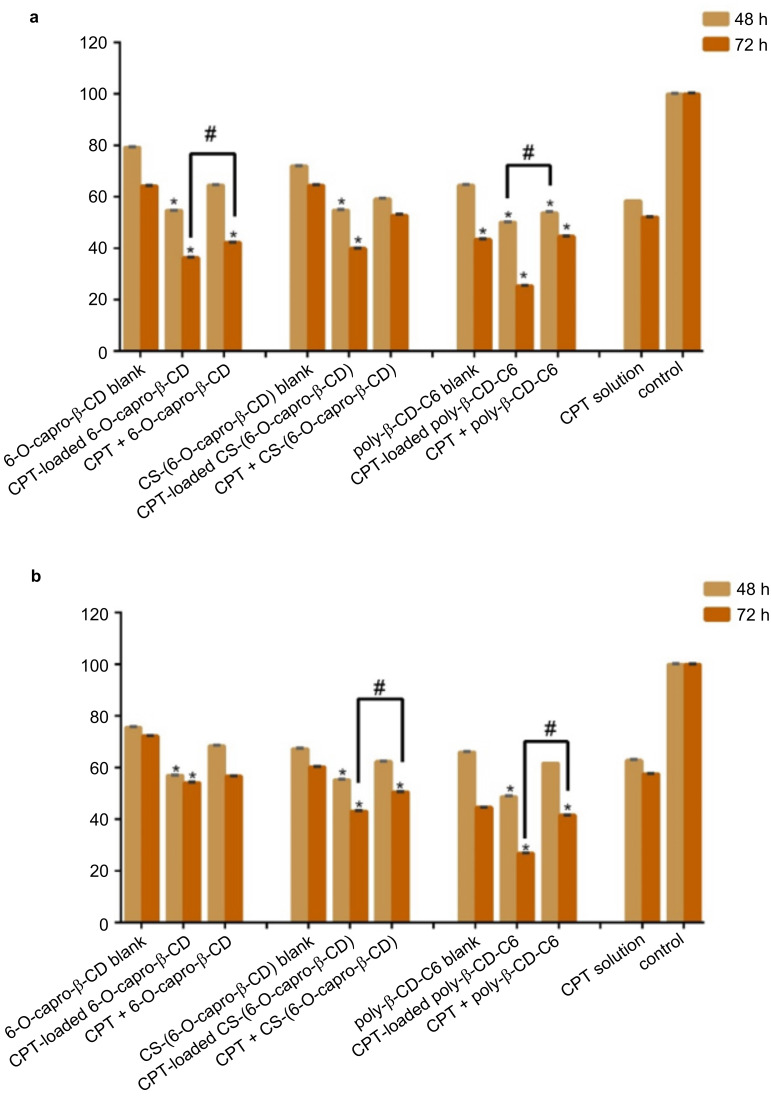
Anticancer effect of blank or drug-loaded cyclodextrin nanoparticles and CPT solution against 3D CT26 murine (a) and HT29 human (b) colon cancer cell line at 48 h and 72 h. Cell viability was evaluated by WST-1 assay. (*n* = 6, mean ± SD), (# *p* < 0.05 and * *p* < 0.05 compared with CPT solution).

According to the results of the anticancer activity analysis performed in the 3D cell culture studies, at the end of the 48-hour incubation period, the effect of drug-loaded CD nanoparticles on cell death was found to be greater than that of the CPT solution on the CT26 cell line (Figure 8a). However, when the 3D spherical tumor results were compared with the 2D conventional cell culture results, significant differences were observed in the efficacy of all drug-loaded CD nanoparticle formulations and each formulation caused more cell death in the 2D cell culture. It was determined that 6-O-capro-β-CD and poly-β-CD-C6 derivatives showed higher anticancer efficacy than drug solution in both drug-loaded nanoparticle formulations and drug + blank nanoparticle formulations that were co-administered. Cell viability was the same for both drug solution and in the group treated with the drug + blank CS-(6-O-capro-β-CD) nanoparticle formulation treated groups at 72 h. In addition, the lowest cell viability was observed in the CPT-loaded poly-β-CD-C6 nanoparticle formulation (Figure 8a). Moreover, cell viability was calculated as 43.5% after 72 hours in the group incubated with blank poly-β-CD-C6 nanoparticle formulations.

Similar results were obtained in the antitumoral efficacy analysis against 3D HT29 spheroids. The most effective formulation were CPT-loaded poly-β-CD-C6 nanoparticles (Figure 8b).

At the end of 72 hours of incubation, there was a significant decrease in cell viability for CS-(6-O-capro-β-CD) and poly-β-CD-C6 derivatives in the drug solution + blank nanoparticle formulation groups compared to the drug solution in 3D HT29 spheroids. Again, it was observed that blank nanoparticles caused a significant decrease in cell viability in 3D cell studies compared to the control group [55,64]. The reduction in cell viability for the blank poly-β-CD-C6 derivative was even higher than for the group that was treated with the drug solution.

As in 2D cell culture results, it was observed that CT26 cells were more sensitive than HT29 cells in cell culture studies with 3D spheroids. It is known that cells in 3D multicellular tumor spheroids typically have lower sensitivity to cytotoxic drugs compared to 2D cultured cells. It is suggested that this difference is due to various reasons, such as decreased drug penetration, development of hypoxic nuclei, and decreased growth [65]. The findings are explained by the differences between conventional cell culture and 3D tumor spheroids. The predicted toxicity/efficacy is enhanced as a result of the formulation's exposure to cells arranged in a monolayer. However, in a 3D cell culture, the disparity in cell proliferation values in mouse and human cell lines is smaller (Figure 8). In terms of formulations, only the 6-O-capro-β-CD nanoparticles have more significant effect on CT26 cells, whilst no other nanoparticle groups show this variation between cell lines.

In 2D conventional cell culture, the cells form a monolayer on the plate and all cells interact equally and directly with the added drug or nanoparticle formulation. Furthermore, it is well recognized that increased intercellular communication by increasing cell–cell contact in 3D cell culture influences drug sensitivity in spherical tumors [66]. In a study, HT29 human colorectal adenocarcinoma cells were treated with an E-cadherin inhibiting antibody before being tested for sensitivity to several anticancer drugs. It was discovered that inhibiting E-cadherins, which are adhesion molecules that provide intracellular connection, increases the sensitivity of 3D colon cancer tumors to 5-fluorouracil, paclitaxel, vinblastine, and etoposide [67]. In the literature, uptake mechanisms of nanoparticles and free drugs in 2D and 3D cell culture methods have been investigated comparatively. The findings showed that nanoparticles and free drugs less effectively reach the underlying cells in 3D spherical tumors due to multilayered cells, and the concentration of 3D tumor-penetrating drugs is lower than in conventional cell culture.

In this paper, cell culture studies were used to evaluate the efficacy of co-administration of drug solution and empty nanoparticles as well as drug-loaded nanoparticles. According to the results of 2D cell culture, the co-administration of CS-(6-O-capro-β-CD) and poly-β-CD-C6 nanoparticles resulted in a significant decrease in cell viability in both cell lines as compared to the drug solution. While there was a significant decrease in CT26 cells in the groups treated with 6-O-capro-β-CD and poly-β-CD-C6 in 3D cell culture, the results for HT29 cells were similar to the conventional 2D cell culture study. As previously noted, CDs have a known affinity for cell membrane structures and components, and they are used for this purpose in the literature. The cholesterol affinity of CD derivatives has been used in the literature for a variety of applications in cancer treatment. Cholesterol concentration has been related to cell membrane fluidity and rigidity, treatment resistance in cancer cells, and drug uptake through the cell membrane. Excipients such as methyl-β-CD are widely used to extract cholesterol from cancer cells such as melanoma and MCF7 cells in order to promote cellular uptake of anticancer medicines [68–70]. It was reported that cholesterol content is related to metastasis and tumor growth in oral squamous cell carcinoma, and cholesterol depletion using methyl-β-cyclodextrin caused an increase in the expression of stem cell markers in cancer cell lines [71]. According to a recent study, cellular cholesterol is directly involved in T cell-mediated cytotoxicity. Cancer cells with cholesterol-rich plasma membranes can evade the immune system by blocking toxicity caused by T cells, but as the amount of cholesterol in the tumor decreases, T cell-mediated cytotoxicity rises [72]. In both traditional and 3D cell culture investigations, incubating cells with empty CD nanoparticles and drug solutions has a synergistic impact due to the antiproliferative activity of the CD nanoparticles themselves. Co-administration of pharmaceuticals with empty nanoparticles, as well as encapsulation into nanoparticles, is an approach worth investigating.

## Conclusion

Oral cancer therapy is still a milestone, attracting researchers' efforts, especially in cancers with high mortality such as CRC. Any progress in this area would be very promising. In this context, oral chemotherapy formulations in the treatment of CRC should be examined comprehensively and in detail, and each past study should shed light on possible future studies. In this study, 3D spheroid tumor models were studied to further elucidate the information we obtained in previous studies, and also mathematical release kinetic modeling was performed for the first time for CPT-loaded amphiphilic cyclodextrin nanoparticles prepared by our team. As a result, when all our publications are evaluated together, it is seen that we have completed comprehensive studies focused on oral CRC treatment with amphiphilic CD nanoparticles. With the increasing progress of studies in this field, it is considered that oral chemotherapy with innovative drug delivery systems in chemotherapy is possible. In this context, especially oral polycationic CD nanoparticles are considered as a promising drug delivery system.

## Experimental

### Materials

**Figure 9:**
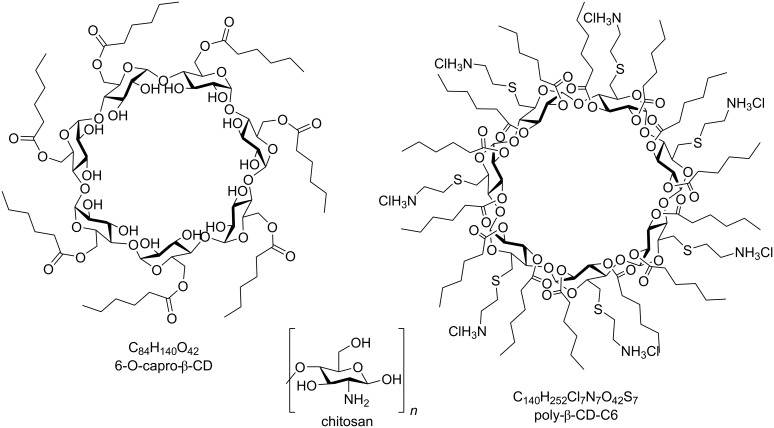
Chemical structures of 6-O-capro-β-CD, poly-β-CD-C6, and chitosan.

6-O-capro-β-CD (MW = 1813 g/mol) and poly-β-CD-C6 (MW = 3178 g/mol) seen in Figure 9 were synthetized, purified, and characterized in the Institute for Chemical Research (CSIC-University of Sevilla, Spain) as previously reported [9,25,50].

(*S*)-(+)-Camptothecin (95% HPLC powder, MW: 348.35 g/mol) was purchased from Sigma-Aldrich, USA. Chitosan (Protasan UP G-113; MW < 200 kDa) was purchased from Novamatrix, Norway. Dialysis cellulose tubing membrane for in vitro release studies (average flat width 25 mm, MWCO: 14,000 Da) was obtained from Sigma-Aldrich, USA. Cell culture studies were performed on CT26 mouse (ATCC^®^ CRL-2638™) and HT29 human colon carcinoma cell line (ATCC^®^ HTB-38™) both purchased from American Type Culture Collection (ATCC, USA). Dulbecco’s modified Eagle medium (DMEM, D5796, Sigma-Aldrich, USA), supplemented with 10% (v/v) fetal bovine serum (FBS, F7524, Sigma-Aldrich, USA) and 1% penicillin/streptomycin (P4333, Sigma-Aldrich, USA) was used for 2D and 3D cell culture studies. Ultrapure water was obtained from a Millipore Simplicity 185 Ultrapure Water System (Millipore, France). All other chemicals were purchased from Sigma-Aldrich and were of analytical purity. Release kinetic analyses were performed using an add-in program, DDSolver 1.0 [73].

### Fabrication and in vitro characterization of CPT-loaded amphiphilic CD NPs

The nanoprecipitation process was used to prepare blank and CPT-loaded nanoparticles, as described previously for amphiphilic CD nanoparticles [9,74]. In earlier investigations, the ideal formulation parameters and component ratios were identified [9,11,74]. Thus, organic solvent (acetone), polycationic CD derivate (0.1% w/v), organic phase:aqueous phase ratio 1:2 (v/v), and 600 rpm stirring rate were applied [9]. In brief, a determined quantity of amphiphilic CD was dissolved in 1 mL of acetone to achieve an organic phase concentration of 0.1% (w/v). This organic phase was added drop-by-drop into 2 mL of the aqueous phase with magnetic stirring at room temperature for 30 min. The organic solvent was evaporated under vacuum at 45 °C to a final dispersion volume of 2 mL. The same technique was used to prepare chitosan (CS)-coated 6-O-capro-β-CD nanoparticles in the presence of CS (0.025% (w/v)) in the aqueous phase. CPT (10% of CD weight) was dissolved in the organic phase to develop drug-loaded nanoparticles. All the details regarding the preparation and characterization of the formulations have been covered extensively in our previous publication [9].

### Drug release from amphiphilic CD nanoparticles

In vitro release experiments were designed to evaluate the release profiles of colon-targeted nanoparticles in the environments encountered along the GIT and the actual transit time. For this purpose, continuous release studies were performed first in pH 1.2 simulated gastric fluid (SGF) within the range of 0–2 hours, then in pH 4.5 simulated intestinal fluid (SIF) during 2–5 hours, and in pH 7.4 simulated colon fluid (SCoF) for the rest of the release period as 5–48 hours, respectively. In this context, the dialysis bag was transferred to the previously prepared release media, respectively [9].

The dialysis membrane method at 37 °C in a shaking water bath (100 rpm) was used. The closed dialysis membrane bag (average flat width 25 mm, MWCO: 14,000 Da) containing the nanoparticle dispersion (3 mL) was then put in release medium (20 mL) that ensured external sink conditions. At predefined time intervals (0.5, 1, 2, 3, 5, 7, 10, and 24 h), 500 µL of sample were taken from the dialysis membrane and replaced with an equal volume of fresh release medium at the same temperature. HPLC was used to quantify the cumulative percentage of CPT released for each time point [9].

### Release kinetics study

In vitro release profiles of CD nanoparticles were analyzed using DDSolver 1.0 [73], designed to reduce computation time and minimize computational errors, and the data were fitted to different kinetic models and analyzed for the appropriate release mechanism (zero order, first order, Higuchi, Korsmeyer–Peppas, Hixson–Crowell, Peppas–Sahlin, Hopfenberg, and Weibull model) [73]. Following the elucidation of the in vitro release profiles of nanoparticles, inputs were computed with the DDSolver software to define the three most important criteria; coefficient of determination (R^2^), Akaike information criterion (AIC), and model selection criterion (MSC). The highest R^2^ and MSC values and the lowest AIC values were used for evaluating different kinetic models [73,75]. Furthermore, release differences or similarities of CPT-loaded amphiphilic cylodextrin nanoparticles were computed according to “difference (*f*1)” and “similarity (*f*2)” factors [73,76] for evaluating through model-independent method. In order to evaluate the release patterns of nanoparticles, the difference factor (*f*1) and similarity factor (*f*2) were computed using a method outlined in the Guidance for Industry from the FDA's Center for Drug Evaluation and Research (CDER) [77]. These two factors can be calculated mathematically by the following equations [78]. *R* and *T* are the percentage dissolved of the reference and test profile, respectively, *t* is the time point, *n* is the number of sampling points. It is noted that *f*1 values for 0–15 and *f*2 values 50–100 indicate that the these release profiles are similar [79].


[2]

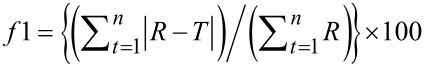




[3] $$f2=50\cdot \log\left[\frac{100}{\sqrt{1+\frac{\sum_{t=1}^{n}{\left(Rt-Tt\right)}^{2}}{n}}}\right]$$

### Cell culture studies

#### Determination of IC_50_ values of camptothecin

IC_50_ values of camptothecin (CPT)-loaded cyclodextrin (CD) nanoparticles and CPT solution in DMSO were determined against CT26 mouse and HT29 human colon carcinoma cell lines at 48 h and 72 h. For this purpose, CT26 and HT29 cells were grown in cell flasks separately. Then, cells were seeded in a 96-well cell culture plate with an initial seeding density of 1 × 10^4^ cells per well in DMEM (100 µL) and allowed to attach overnight. The formulations were diluted with serum-free DMEM to obtain the appropriate CPT concentration according to their loading efficiency, and the medium on the cells was replaced with medium containing the formulation and incubated for 48 and 72 hours. Our previous research demonstrated that HT29 cells treated with 1.44 µM CPT after 48 hours of incubation had almost 50% viability [9]. Therefore, the lower and upper concentrations of CPT (0.4, 0.8, 1.6, 3.2, 6.4, and 12.8 µM) were examined in this study. Equal DMSO concentrations were applied to the cells as a separate group, and cell viability was normalized relative to the DMSO group. After 48 h and 72 h of incubation time, cell viability was determined by the WST-1 assay with a microplate reader at a wavelength of 450 nm. Cells that were incubated with the medium were used as control group with 100% cell viability. The following equation was used to calculate cell viability percentage


[4] $$\begin{array}{l} \text{Cell viability (}\%)=\\ \frac{\text{optical density (OD) of treated cells}}{\text{optical density (OD) of untreaded cells}}\times \text{100} \end{array}$$

IC_50_ values were calculated with the GraphPad Prism version 6 (San Diego, CA, USA) using the data of cell viability against increasing drug concentration.

#### Determination of doubling time

The initial cell number concentration was calculated to use the same number of cells in 2D and 3D cell culture studies. For this purpose, first the doubling times of the cells were determined. Separately, CT26 and HT29 cells were seeded into 96-well plates (10,000 cells/well). After 48 hours of incubation, the medium was removed from the plates, cells were trypsinized and counted. Based on the formula below, the doubling time was calculated.


[1] $$\begin{array}{l} \text{Doubling time}=\\ \frac{\text{duration}*\log(2)}{\log(\text{final concentration})-\log(\text{initial concentration})} \end{array}$$

#### Evaluation of anticancer efficiency of CPT-loaded CD nanoparticles on 2D cell cultures

The anticancer activities of CPT-loaded amphiphilic CD nanoparticles were determined against CT26 and HT29 cell lines. Cells were seeded at a density of 1 × 10^4^ cells/well in full DMEM (100 µL) into each well of 96-well plates. The cells were then cultured at 37 °C for 24 h in a 5% CO_2_ incubator. The medium was replaced after 24 h with new serum-free medium containing drug solution, blank nanoparticle formulations, drug-loaded nanoparticles (CPT-loaded 6-O-capro-β-CD, CPT-loaded CS-(6-O-capro-β-CD), and CPT-loaded poly-β-CD-C6), or free drug solution + blank nanoparticle formulations (CPT + 6-O-capro-β-CD, CPT + CS-(6-O-capro-β-CD), and CPT + poly-β-CD-C6). After 48 h and 72 h of incubation, WST-1 (10 μL) was added to the cells. After a 3-hour incubation period, the absorbance at 450 nm was measured using a microplate reader, and cell viability was estimated. A viability/cytotoxicity assay kit was also used to test cell viability microscopically (30002, Biotium, Fremont, CA, USA). CT26 and HT29 cells were incubated with nanoparticle formulations or free drug solution for 48 h. Then, the medium was removed, and 200 µL of dye mixture were added to each well and incubated for 45 minutes further. Fluorescence microscopy was used to see groups of cells after the incubation.

#### Evaluation of antitumoral efficiency of CPT-loaded CD nanoparticles on 3D cell cultures

The scaffold-based approach for in vitro 3D cell culture studies, which was previously reported by Varan et al. [80], was adopted for this paper. Poly(2-hydroxyethyl methacrylate) (poly-HEMA, P3932, Sigma, St. Louis, MO, USA) was used to achieve a low attachment surface in round-bottomed wells. Under sterile conditions, 1.2 g of poly-HEMA was dissolved in 40 mL of 95% ethanol, and 50 µL of this solution were distributed into each well. For at least 24 hours, plates were maintained under laminar flow to evaporate the organic solvent. Following this evaporation, CT26 or HT29 cells (1,250 cells/200 µL medium per well) were added into each well in DMEM containing 3% Matrigel^®^ Basement Membrane Matrix, and the plate was agitated at 1,000 rpm for 10 minutes. 100 µL of fresh medium was replaced every 2 days. Microscopical analysis of the spheroid development was performed. After 3 days, DMEM was exchanged with nanoparticle formulations, and cell viability was measured at 48 h and 72 h using the WST-1 assay.
